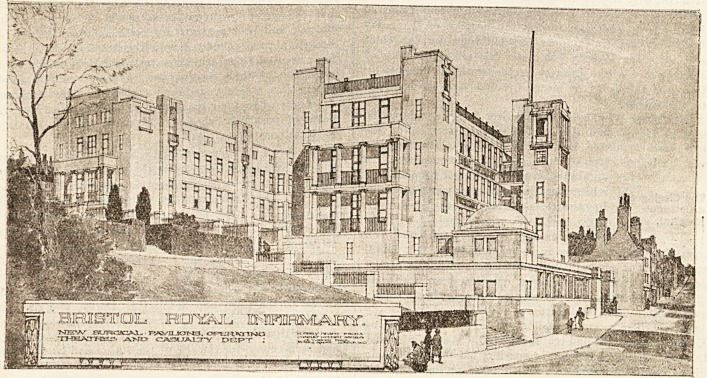# Eminent Chairmen Series
*The previous articles in this series appeared in The Hospital of Oct. 1, Nov. 1, Dec. 10, Jan. 14, Feb. 11 March 11, and April 22.


**Published:** 1911-05-20

**Authors:** 


### SPECIAL INSTITUTIONAL ARTICLE.

### EMINENT CHAIRMEN SERIES.*

### VIII.—SIR GEORGE WHITE, Bart., President and Treasurer of Bristol Royal Infirmary.

THE President and Treasurer of Bristol's oldest medical charity, the Bristol Royal Infirmary, the subject of our article this week, is Sir George White, Bart., who was elected to the post in 1904.

Sir George, who is a Bristolian, is the head of the firm of George White and Co., and a Past President of the Bristol Stock Exchange as well as of the Council of Associated Stock Exchanges of the United Kingdom. He is a J.P. for Bristol, one of the Bristol Municipal Charity Trustees, a Governor of the Bristol Grammar and other schools, Chairman of several important undertakings in Bristol and South Wales, and President of the Queen Victoria Memorial Hospital at Nice. He was the first to introduce electric street traction in London, Dublin, Bristol, Middlesbrough, etc. Last year he entered the aviation world as Chairman of the British and Colonial Aeroplane Company, the constructors of the already noted "Bristol" aeroplanes.

SIR GEORGE WHITE, BART., PRESIDENT AND TREASURER BRISTOL ROYAL INFIRMARY.

FIRST ACTIVITIES.

It was in 1904 that the Committee and Governors of the Bristol Royal Infirmary invited Sir George to become the President and Treasurer of the institution, which was then burdened with a heavy revenue deficit of £15,552, whilst a large sum of money was required to deal with the very pressing necessity of renewing and restoring the fabric. The President's first objective was to raise a £50,000 fund which would enable the institution to clear off its revenue debt of £15,552 and provide nearly £35,000 for the purpose of making the alterations and improvements of which the Infirmary stood so badly in need. With this end in view a Carnival was organised on a huge scale at the Clifton Zoological Gardens in June 1905. It was an unqualified success, and produced a profit of £8,038 (including £4,015 contributed by Mr. Samuel White, Sir George's brother) and Sir George himself then gave the £7,514 to enable the deficit of £15,552 to be entirely cleared off, and the institution was able to make a fresh start with a clean sheet.

THE RENOVATION SCHEME.

Having dealt with the revenue position, Sir George turned his attention to raising the amount required for the renovation and alteration of the buildings, and within the twelve months he was able to announce that the amount of £34,448 necessary to complete the £50,000 fund had been subscribed by the citizens of Bristol and surrounding districts. The President and Committee then devoted a large amount of time to dealing with the question of the extension of the Institution, and after considering various schemes for the enlargement and improvement of the old building, finally decided to construct a new Surgical Infirmary on a site which had been purchased on the opposite side of the road.

* The previous articles in this series appeared in THE HOSPITAL of Oct. 1, Nov. 1, Dec. 10, Jan. 14, Feb. 11 March 11, and April 22.

They formulated their requirements and arranged a public competition amongst architects, with the result that the plans laid before them by Mr. Percy Adams, F.R.I.B.A., were awarded the first prize. The actual work of clearing the site was commenced last July, the foundation stone being laid by Sir George on March 14 last, and it is hoped to have the building completed by March next. We give in this issue a reproduction of a drawing of the new Surgical Infirmary as it will appear when completed, providing for a fine suite of operating theatres, five surgical wards of 24 beds each, three of eight beds, one of twelve and one of ten beds, five of two beds and five special one-bed wards, making in all 181 beds, as well as large and up-to-date casualty and receiving rooms, with a spacious entrance hall and excellent lifts. The external elevation will be of an unusually simple and dignified design, being carried out on all sides in white Portland stone, the whole being connected with the old Infirmary by a wide subway. The scheme, which represents an expenditure of £70,000, was cordially approved by a special Board of Governors in June last, at which meeting a suggestion was made that this new surgical block would form a fitting memorial to the late King Edward. His Majesty King George has recently given his gracious consent to the extension being known as the "King Edward Memorial Infirmary."

The Bristol Royal Infirmary.

The Royal Infirmary, which was established in 1735, claims to be the oldest medical charity supported entirely by voluntary contributions in the provinces, and is the largest general hospital in the West of England. When Sir George entered into the active management of the Institution in 1904 the annual subscription list totalled £3,187, received from 1,290 subscribers, but his endeavours to place the finances upon a sound basis have resulted in practically doubling the subscription list during the six years intervening, the accounts for last year showing that 3,075 subscribers contributed £6,062 as annual subscriptions to the institution, an increase of £2,875. Sir George is not satisfied with only increasing the income of the Institution, but he has also turned his attention to the expenditure side of the accounts, with the result that during his tenure of office he has been able to exercise such economies, consistent with greater efficiency in the working of the Institution, as to reduce the cost of each occupied bed from £62 6s. 11d. in 1904 to £55 10s. 1d. in 1910, and this notwithstanding that the resident medical and surgical staff of the Infirmary has during that period been greatly increased. The cost of each in-patient has also been reduced from £3 18s. to £3 4s. 2d. in the same period.

Special Departments.

Various special departments, such as the Ear, Throat, and Nose, and the Pathological, have been developed, and the Massage Department has been started since Sir George White became President. The fame of the Bristol Royal Infirmary is such, extending over the whole of the West of England, that there is increasing difficulty in coping with the vast number of patients presenting themselves for treatment. The wards are constantly crowded and there are long waiting lists in the various departments. Last year the Institution treated 4,149 in-patients and 52,813 out-patients, or a total of 56,962, which is the largest number admitted in any one year since the foundation of the Infirmary, whilst since it opened its doors in 1735 it has ministered to the wants of 2,376,220 sick and suffering men, women, and children.

The Nursing Department has been raised to the highest state of efficiency, and during Sir George's presidency one of the earliest preliminary training schools for nurses has been started, the two years of its work having been highly satisfactory. Fifty-six probationers a year pass through its preparation, and already the accommodation provided is proving inadequate for the number of probationers required for the service of the Institution. The private nursing staff, consisting of nurses all of whom have received three years' training in the wards of the Bristol Royal Infirmary, has been more than doubled and their earnings have largely increased the finances of the Institution, but the nurses on the private nursing staff may, after a stipulated time, join the Private Nurses' Co-operation, in which case they receive the whole of their earnings with the exception of a small percentage retained for administrative and other expenses. The President takes a special pride in the deservedly high reputation of the nurses trained in the Bristol Royal Infirmary. The number of matrons and other administrative posts recently obtained by the sisters of this infirmary is very gratifying to all concerned.

From the scanty particulars here given it will be seen that Sir George White has undertaken an enormous task, but those who are working with him have absolute faith in their leader, and know that the result of his labours will be the completion of an infirmary fulfilling its ancient traditions and worthy of the city to which it belongs.

Sir George does not confine his work in connection with medical charities to these shores alone, but, as mentioned, is also President of the Queen Victoria Memorial Hospital at Nice, which, as its name implies, was erected as a memorial to her late Majesty by the English-speaking residents on, and visitors to, the Riviera. In 1905, on the death of Sir Blundell Maple, Bart., who was the President of the Memorial Association, the completion of the memorial received an unexpected check in consequence of the executors of the late Sir Blundell being unable at first to obtain permission from the Law Courts to pay out of the estate funds the donation of £3,000 promised by him. For a time it appeared that the Association would be unable to complete the memorial, but Sir George, hearing of the dilemma, gave a donation of £3,000 so as to carry on the work to completion. He was elected President in the late Sir Blundell's stead, and later on, as the result of renewed application to the Courts, Sir Blundell's donation of £3,000 was also paid, much to the advantage of the hospital. In March 1906 the hospital, which exists solely for the use of British subjects and American citizens, without distinction of creed, was opened by H.R.H. Princess Christian of Schleswig-Holstein, since when it has dealt with a large number of serious cases, many of the patients being in extremely straitened circumstances, and has, by alleviating the suffering of many British and American persons, without doubt more than justified its establishment. The hospital, including the isolation annexe presented by Sir Henry B. Samuelson, Bart, now contains 50 beds, the majority of which are entirely free for necessitous cases. The original conception of the founders of the Queen Victoria Memorial was to erect what is known in England as a cottage hospital, at a cost of about £3,000, but since Sir George took over the presidency it has been found necessary from time to time to enlarge the structure so that at the present time there has been spent upon the building and equipment about £25,000, all of which has been subscribed by the English and American residents and visitors.

## Figures and Tables

**Figure f1:**
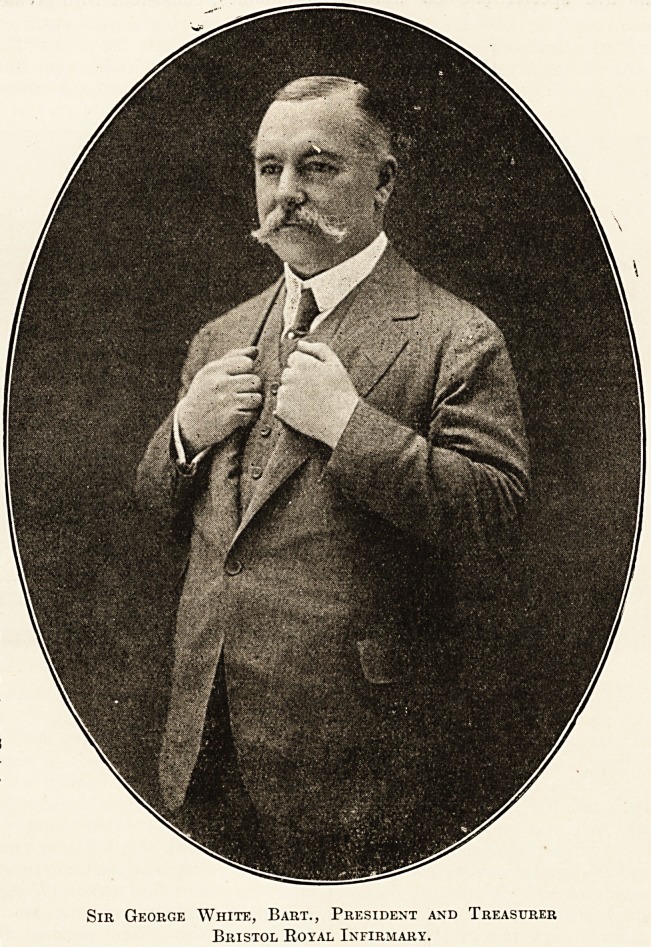


**Figure f2:**